# Deletion of Inducible Nitric-Oxide Synthase in Leptin-Deficient Mice Improves Brown Adipose Tissue Function

**DOI:** 10.1371/journal.pone.0010962

**Published:** 2010-06-04

**Authors:** Sara Becerril, Amaia Rodríguez, Victoria Catalán, Neira Sáinz, Beatriz Ramírez, María Collantes, Iván Peñuelas, Javier Gómez-Ambrosi, Gema Frühbeck

**Affiliations:** 1 Metabolic Research Laboratory, Clínica Universidad de Navarra, Pamplona, Spain; 2 Department of Endocrinology, Clínica Universidad de Navarra, Pamplona, Spain; 3 CIBER Fisiopatología de la Obesidad y Nutrición, Instituto de Salud Carlos III, Madrid, Spain; 4 MicroPET Research Unit CIMA-CUN, University of Navarra, Pamplona, Spain; University of Las Palmas de Gran Canaria, Spain

## Abstract

**Background:**

Leptin and nitric oxide (NO) on their own participate in the control of non-shivering thermogenesis. However, the functional interplay between both factors in this process has not been explored so far. Therefore, the aim of the present study was to analyze the impact of the absence of the inducible NO synthase (*iNOS*) gene in the regulation of energy balance in *ob/ob* mice.

**Methods and Findings:**

Double knockout (DBKO) mice simultaneously lacking the *ob* and *iNOS* genes were generated, and the expression of molecules involved in the control of brown fat cell function was analyzed by real-time PCR, western-blot and immunohistochemistry. Twelve week-old DBKO mice exhibited reduced body weight (*p<*0.05), decreased amounts of total fat pads (*p<*0.05), lower food efficiency rates (*p<*0.05) and higher rectal temperature (*p<*0.05) than *ob/ob* mice. Ablation of *iNOS* also improved the carbohydrate and lipid metabolism of *ob/ob* mice. DBKO showed a marked reduction in the size of brown adipocytes compared to *ob/ob* mutants. In this sense, in comparison to *ob/ob* mice, DBKO rodents showed an increase in the expression of PR domain containing 16 (*Prdm16*), a transcriptional regulator of brown adipogenesis. Moreover, *iNOS* deletion enhanced the expression of mitochondria-related proteins, such as peroxisome proliferator-activated receptor γ coactivator-1 α (Pgc-1α), sirtuin-1 (Sirt-1) and sirtuin-3 (Sirt-3). Accordingly, mitochondrial uncoupling proteins 1 and 3 (Ucp-1 and Ucp-3) were upregulated in brown adipose tissue (BAT) of DBKO mice as compared to *ob/ob* rodents.

**Conclusion:**

Ablation of *iNOS* improved the energy balance of *ob/ob* mice by decreasing food efficiency through an increase in thermogenesis. These effects may be mediated, in part, through the recovery of the BAT phenotype and brown fat cell function improvement.

## Introduction

Energy homeostasis is a highly regulated process that requires a tight balance between caloric intake and energy expenditure [1]. The latter is a key determinant of energy balance and includes three components: basal metabolic rate, physical activity, and adaptive thermogenesis [2], [3]. In this sense, brown adipose tissue (BAT) constitutes a highly active metabolic organ that plays a crucial role in non-shivering thermogenesis, defined as the heat production in response to cold or overfeeding [4]. Until recently, BAT was thought to be important only in small mammals and newborn humans. However, functional BAT was recently identified in adults, suggesting a role in human metabolism [5], [6]. In brown adipocytes, thermogenesis is mainly mediated by sympathetically innervated β_3_-adrenergic receptors, leading to the activation of the BAT-specific uncoupling protein-1 (Ucp-1). This protein is a proton transporter located in the inner mitochondrial membrane that diverts the energy from the mitochondrial respiratory chain from ATP synthesis to heat production [7]. The *Ucp-1* promoter is regulated by several transcriptional coactivators, including the peroxisome proliferator-activated receptor γ (PPARγ) coactivator-1 α (Pgc-1α), being also involved in the regulation of crucial aspects of energy metabolism [8], [9]. Pgc-1α is strongly induced in murine BAT during cold exposure activating the thermogenic gene program of brown fat through the control of the gene expression levels of *Ucp-1* and *Pgc-1α* itself. In this regard, it has been recently described that during BAT differentiation PR domain containing 16 (Prdm16) directly binds to Pgc-1α, allowing the activation of *Ucp-1* and other brown fat-specific genes [10], [11]. Moreover, it has been demonstrated that the NAD^+^-dependent deacetylase sirtuin-1 (Sirt-1) deacetylates and activates Pgc-1α in the liver and BAT [12], [13], allowing its union to target genes and increasing the rate of gene transcription. The key role of the correpresor of nuclear receptor-interacting protein 1 (*Nrip*), also known as receptor-interacting protein 140 (*Rip140*), in energy homeostasis by suppressing the transcription of *Ucp-1* and other metabolic genes has been also reported [14], [15].

Leptin, the product of the *ob* gene, plays a key role in the control of body weight by suppressing food intake through actions on hypothalamic receptors and by increasing energy expenditure via the activation of the sympathetic nerve activity and the turnover of norepinephrine in BAT [16], [17]. Leptin induces the gene expression of *Pgc-1α* and *Ucp-1* through the stimulation of β_3_-adrenergic receptors, thereby leading to an increased thermogenesis [18]–[21]. In this sense, it has been shown that leptin-deficient *ob/ob* mice are obese, hyperphagic and exhibit reduced non-shivering thermogenesis as well as low UCP-1 levels in BAT [22].

Previous studies showed that norepinephrine increases the blood flow in BAT by stimulating the production of nitric oxide (NO), a potent vasodilator [23]. NO is produced by NO synthase (NOS), and three isoforms have been identified: the endothelial (eNOS) and neuronal (nNOS), which are constitutively expressed, together with the inducible NOS (*iNOS*), which is primarily transcriptionally regulated by immunologic as well as inflammatory stimuli [24]. Both eNOS and *iNOS* isoforms have been shown to be expressed in brown adipocytes [25], providing evidence for the involvement of NO in BAT function regulation.

The deletion of the *iNOS* gene reportedly prevents high-fat diet-induced insulin resistance [26]. Furthermore, leptin and *iNOS* on their own participate in multiple common physiological processes, with a functional relationship between both factors having been described earlier by our group [27]–[29] and others [30], [31]. In order to explore the functional interplay between both factors and to better understand the regulatory pathways that govern energy metabolism, we examined the effects of *iNOS* gene disruption in genetically obese *ob/ob* mice on the diverse elements of energy balance focusing particularly on the expression of non-shivering thermogenesis-related molecules. Our study shows that deletion of the *iNOS* gene decreases food efficiency through an increase in thermogenesis, thus improving the energy balance of *ob/ob* mice.

## Materials and Methods

### Generation of double-knockout mice lacking the *ob* and *iNOS* genes

A double knockout (DBKO) mouse simultaneously lacking the *ob* and the *iNOS* genes was generated by intercrossing male *ob/ob* mice with female *iNOS* knockout mice (*iNOS*−^/^−) on a C57BL/6J background (Jackson Laboratories, Bar Harbor, ME, USA). Noteworthy, o*b/ob* male mice were placed under caloric restriction (2 g standard chow diet/day) and were daily injected with recombinant leptin (2 g/kg body weight) (Bachem, Bubendorf, Switzerland) in order to overcome the infertility problems of the leptin-deficient rodents [32]. Genotyping for *ob* and *iNOS* was performed as previously described [32], [33]. Briefly, genomic DNA was extracted from ear clips by using the DNeasy Mini kit (Qiagen, Valencia, CA, USA) according to the manufacturer's instructions. The genotyping strategy utilized the *Dde I* restriction site generated by the *ob* mutation. To identify the presence of the wild type or the disrupted *iNOS* allele, three different primers were used. One primer complementary to both the genomic and disrupted alleles that amplified a 108-bp fragment with the second primer specific for the genomic sequence and a 275-bp fragment with the third primer complementary to a region of the neomycin resistance insert specific to the disrupted *iNOS* allele [34]. The PCR was performed as described elsewhere [33] and the PCR products were separated on a 1.5% agarose gel and visualized with ethidium bromide staining.

Male mice were weaned at 21 days of age, genotyped, and maintained at a room temperature of 22±2°C on a 12∶12 light-dark cycle (lights on at 08:00 am) with a relative humidity of 50±10% and under pathogen-free conditions. Animals had free access to tap water and were fed *ad libitum* with a normal chow diet (2014S Teklad Global 14% Protein Rodent Maintenance Diet, Harlan, Barcelona, Spain). Body weight and food intake were registered twice weekly. The food efficiency was determined as body weight gained per week divided by total energy (kilocalories) consumed over this period [35]. Body temperature was assessed by measuring rectal temperature using a rectal thermoprobe (YSI 4600 Series Precision Thermometers, YSI Temperature, Dayton, OH, USA). The diameter of adipocytes was determined by direct microscopy, and the cell size was obtained using digital photographs with the *Axiovision 4.6 program* (Zeiss). Mice were injected in the tail vein with ^18^F-fluorodeoxyglucose (^18^F-FDG) (200 µCi) after a short isoflurane (4%) anesthesia period. The uptake of ^18^F-FDG was analyzed by a Mosaic (Philips Electronics, Amsterdam, The Netherland) small-animal dedicated imaging tomograph as previously described [36].

### Blood and tissue collection

Twelve-week-old mice were fasted for 6 hours and sacrificed by CO_2_ inhalation. Blood samples were obtained by cardiac puncture, and sera collected after cold centrifugation (4°C) at 700 *g* for 15 min and stored at −20°C. Epididymal, subcutaneous and perirenal white adipose tissue together with brown fat from the interscapular depots were carefully excised. Tissue samples were immediately frozen at −80°C. Biopsies of BAT were also formalin-fixed for immunohistochemical analyses. All experimental procedures conformed to the European Guidelines for the Care and Use of Laboratory Animals (Directive 86/609) and the study was approved by the Ethical Committee for Animal Experimentation of the University of Navarra (042/03, 041/08).

### Blood measurements

An intraperitoneal glucose tolerance test was performed after an overnight fasting period (12 h). Mice were injected intraperitoneally with glucose (2 g/kg of body weight). Glucose concentrations were measured before and 15, 30, 60, 90 and 120 min after the glucose challenge. Glucose was determined by an automatic glucose sensor (Ascensia Elite, Bayer, Barcelona, Spain) from whole blood obtained from the tail vein. Serum glucose was measured by a glucometer (Ascensia Elite). Serum concentrations of triglycerides, total cholesterol (Infinity, Thermo Electron Corporation, Melbourne, Australia), free fatty acids (FFA) (Wako Chemicals, GmbH, Neuss, Germany) and glycerol (Sigma, St. Louis, MO, USA) were measured by enzymatic methods using commercially available kits. Insulin and adiponectin were determined by ELISA (Crystal Chem, Inc., Chicago, IL, USA and BioVendor Laboratory Medicine, Inc., Modrice, Czech Republic, respectively). Intra- and inter-assay coefficients of variation for measurements of insulin and adiponectin were 3.5% and 6.3%, respectively, for the former, and 5.6% and 7.2%, for the latter.

### Western blot studies

Tissues were homogenized and protein content was measured as described earlier [37]. Equal amounts of protein (30 µg) were run in 8% SDS-PAGE, subsequently transferred to nitrocellulose membranes (Bio-Rad Laboratories, Inc., Hercules, CA, USA), and blocked in Tris-buffered saline (TBS) with Tween 20 containing 5% non-fat dry milk for 1 h at room temperature (RT). Blots were then incubated overnight at 4°C with primary antibodies against Ucp-1 and Ucp-3 (Abcam) at 1∶10,000 and 1∶8,000, respectively; Pgc-1α (Cell Signalling Technology, Inc., Danvers, MA, USA) at 1∶1,000; Sirt-1 (Abcam Ltd., Cambridge, UK) at 1∶1,000; Sirt-3 (Cell Signalling Technology, Inc) at 1∶1,000; or β-actin (Sigma) at 1∶5,000. The antigen-antibody complexes were visualized using peroxidase-conjugated anti-rabbit or anti-mouse IgG antibodies (1∶5,000) and the enhanced chemiluminescence ECL detection system (Amersham Biosciences, Buckinghamshire, UK). The intensity of the bands was determined by densitometric analysis with the *Gel Doc^TM^* gel documentation system and the *Quantity One* 4.5.0 software (Bio-Rad) and normalized with β-actin densitometric values. All assays were performed in duplicate.

### Immunohistochemistry of Ucp-1 and Ucp-3

The immunohistochemistry was carried out using the indirect immunoperoxidase method. Sections (6 µm) of formalin-fixed paraffin-embedded BAT were dewaxed in xylene, rehydrated in decreasing concentrations of ethanol and treated with 3% H_2_O_2_ (Sigma) in absolute methanol for 10 min at RT to quench endogenous peroxidase activity. Then, sections were immersed in 10 mmol/l citrate buffer (pH 6.00) and heated using a microwave oven at 800 W for 15 min to enhance antigen retrieval. After cooling, slides were blocked during 1 h with 1% murine serum (Sigma) diluted in Tris-buffer saline (TBS) (50 mmol/l Tris, 0.5 mol/l NaCl; pH 7.36) for preventing non-specific adsorption. Sections were incubated overnight at 4°C with rabbit monoclonal anti-Ucp-1 or Ucp-3 antibodies (Abcam) diluted 1∶100 in TBS. After three washes (5 min each) with TBS, sections were incubated with horseradish peroxidase-conjugated anti-rabbit IgG antibody (Amersham Biosciences) diluted 1∶200 in TBS for 30 min at RT. After washing in TBS, the peroxidase reaction was visualized with a 3,3′-diaminobenzidine (DAB, Amersham Biosciences)/H_2_O_2_ solution (0.5 mg/ml DAB, 0.03% H_2_O_2_ diluted in 50 mmol/l Tris-HCl, pH 7.36) as chromogen, and Harris hematoxylin solution (Sigma) as counterstaining. Sections were dehydrated, coverslipped and observed under a Zeiss Axiovert 40 CFL optic microscope (Zeiss, Göttingen, Germany). Negative control slides without primary antibody were included for the assessment of non-specific staining.

### RNA extraction and Real-Time PCR

Total RNA was extracted from BAT samples by homogenization with an ULTRA-TURRAX® T 25 basic (IKA® Werke GmbH, Staufen, Germany) using TRIzol® Reagent (Invitrogen, Barcelona, Spain). Samples were purified with the RNeasy Mini kit (Qiagen) according to the manufacturer's instructions and treated with DNase I (RNase-free DNase Set, Qiagen). For first strand cDNA synthesis constant amounts of 2 µg of total RNA were reverse transcribed in a 40 µl final volume using random hexamers (Roche Molecular Biochemicals, Mannheim, Germany) as primers and 400 units of M-MLV reverse transcriptase (Invitrogen) as described earlier [38].

The transcript levels for genes involved in brown fat cell differentiation and function (*Prdm16*, *Rip140*, *Bmp7*, *Sirt-1*, *Sirt-3*, *Pgc-1α*, *Ucp-1* and *Ucp-3*) were quantified by Real-Time PCR (7300 Real Time PCR System, Applied Biosystems, Foster City, CA, USA). Primers and probes were designed using the software *Primer Express 2.0* (Applied Biosystems) (Table 1) and purchased from Genosys (Sigma). TaqMan® probes encompassing fragments of areas from the extremes of two exons were designed to ensure the detection of the corresponding transcript avoiding genomic DNA amplification. The cDNA was amplified at the following conditions: 95°C for 10 min, followed by 45 cycles of 15 s at 95°C and 1 min at 59°C, using the TaqMan® Universal PCR Master Mix (Applied Biosystems). The primer and probe concentrations for gene amplification were 300 nmol/l and 200 nmol/l, respectively. All results were normalized to the levels of *18S* rRNA (Applied Biosystems) and relative quantification was calculated using the ΔΔCt formula [38]. Relative mRNA expression was expressed as fold expression over the calibrator sample (average of gene expression corresponding to the wild type group) [39]. All samples were run in triplicate and the average values were calculated.

**Table 1:** Sequences of the primers and TaqMan® probes.

Gene (GenBank accession number)	Oligonucleotide sequence (5′-3′)
*Prdm16* (NM_027504)	
Forward	GATGGGAGATGCTGACGGATAC
Reverse	CTCGCTACCCAAGTCTTCAGAGAT
TaqMan® Probe	FAM- CATCCCAGGAGAGCTGATCAAAAAGC-TAMRA
*Rip140* (NM_173440)	
Forward	TCAGCTTCCTTTCCCACATAGC
Reverse	TCATCTTTCGTTGCTCACCAAA
TaqMan® Probe	FAM-AGGCTCAGGCTGAGGCAGACGATACT-TAMRA
*Bmp7* (NM_07557)	
Forward	CAAGACGCCAAAGAACCAAGAG
Reverse	GGTCTCGGAAGCTGACGTACAG
TaqMan® Probe	FAM-ATGGCCAGTGTGGCAGAAAACAGCA-TAMRA
*Sirt-1* (NM_019812)	
Forward	AGCAGGTTGCAGGAATCCAA
Reverse	CACGAACAGCTTCACAATCAACTT
TaqMan® Probe	FAM-CCTTCAGTGTCATGGTTCCTTTG-TAMRA
*Sirt-3* (NM_001127351)	
Forward	CTGACTTCGCTTTGGCAGATCT
Reverse	CCCCACCAAGTCTCGATTGAT
TaqMan® Probe	FAM-CTGGAGGTGGAGCCTTTTGCCAGCT-TAMRA
*Pgc-1α* (NM_008904)	
Forward	TGAACGCACCTTAAGTGTGGAA
Reverse	GGGTTATCTTGGTTGGCTTTATGA
TaqMan® Probe	FAM-ATCGCAGGCCTAACTCCACCCACCA -TAMRA
*Ucp1* (NM_009463)	
Forward	CGATGTCCATGTACACCAAGGA
Reverse	ACCCGAGTCGCAGAAAAGAAG
TaqMan® Probe	FAM-ACCGACGGCCTTTTTCAAAGGGTTTG-TAMRA
*Ucp3* (NM_001030877)	
Forward	GACCTACGACATCATCAAGGAGAAGT
Reverse	CTCCAAAGGCAGAGACAAAGTGA
TaqMan® Probe	FAM-6TCTCACCTGTTTACTGACAACTTCCC-TAMRA

*Prdm16*, PR domain containing 16; *Rip140*, receptor-interacting protein 140; *Bmp7*, bone morphogenetic protein 7; *Sirt-1*, sirtuin-1, *Sirt-3*, sirtuin-3; *Pgc-1α*, peroxisome proliferative activated receptor γ coactivator 1 α; *Ucp-1*, uncoupling protein 1, *Ucp-3*, uncoupling protein 3.

### Statistical analysis

Data are presented as the mean ± SEM. Differences between groups were assessed by two-way ANOVA. In case of interaction between factors (lack of *iNOS* or *ob* genes), one-way ANOVA followed by Tukey's *post hoc* tests were applied. Statistics were calculated by the SPSS/Windows version 15.0 software (SPSS, Inc., Chicago, IL, USA). A P value less than 0.05 was considered statistically significant.

## Results

### Ablation of *iNOS* reduces the positive energy balance of *ob/ob* mice

**Figure 1:**
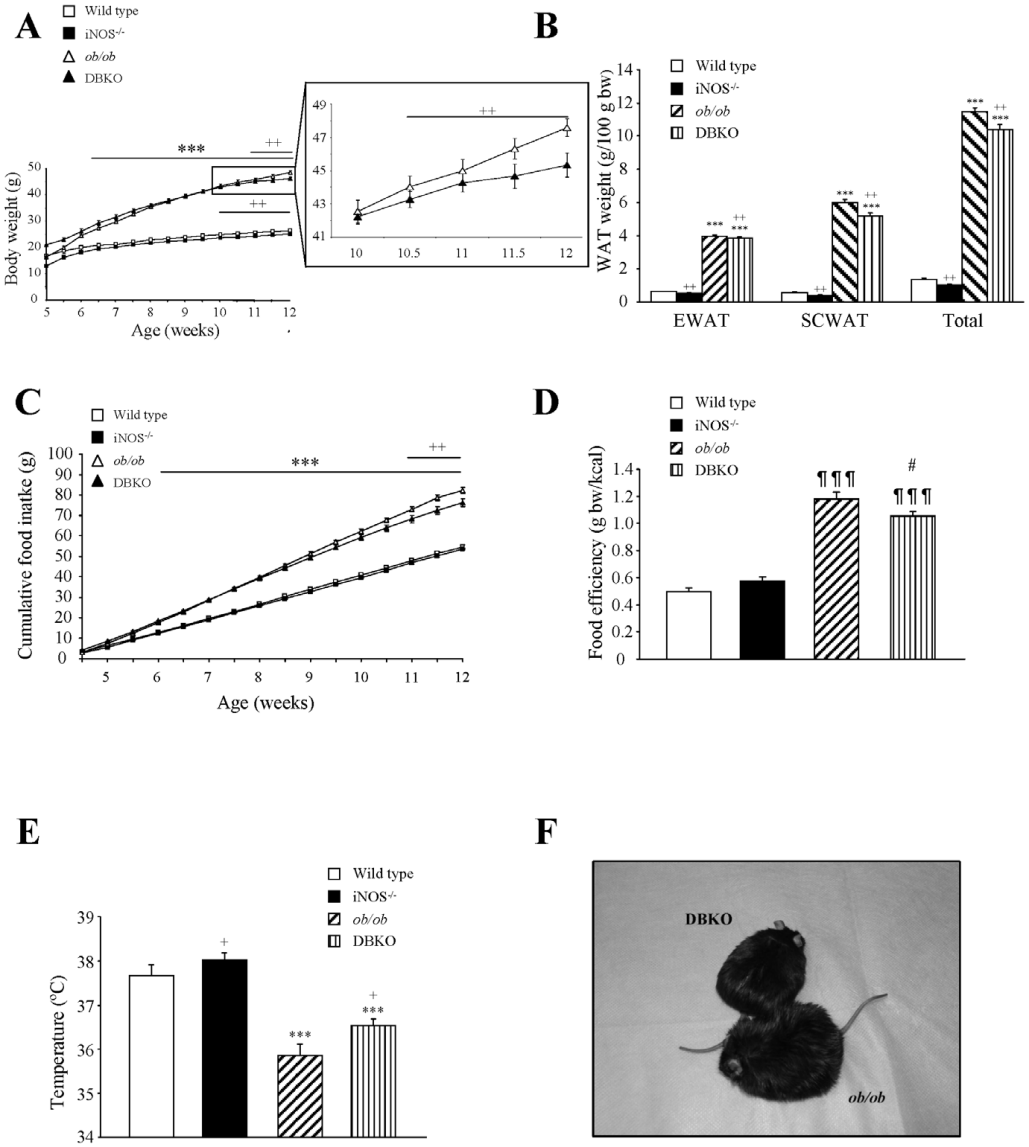
Growth and metabolic variables of mice of the four experimental groups. Growth curves of 4–12 week-old-mice (A) together with epididymal (EWAT), subcutaneous (SCWAT) and whole-body fat content (B) of the experimental animals. Cumulative food intake (C), food efficiency (D) and rectal temperature (E) are also shown. Representative images illustrating the differences in size between 12-week-old *ob/ob* and DBKO mice (F). Values are the mean ± SEM (n = 10 per group). Differences between groups were analyzed by two-way ANOVA. ***p<0.001, effect of the absence of the *ob* gene. +*p<*0.05, ++*p<*0.01, effect of the absence of the *iNOS* gene. One-way ANOVA followed by Tukey's *post hoc* test was applied for variables with interaction between factors. ¶¶¶*p<*0.001 vs wild type, #*p<*0.05 vs *ob/ob* mice. EWAT, epididymal white adipose tissue; SCWAT, subcutaneous white adipose tissue; WAT, white adipose tissue; bw, body weight.

As expected, leptin deficiency was associated with increased (*p<*0.001) body weight, higher fat depots, hyperphagia and hypothermia, whereas *iNOS* deficiency resulted in reduced (*p<*0.01) body weight and lower fat content as well as increased (*p<*0.01) body temperature as compared to control mice (Fig. 1). The *ob/ob* mice lacking the *iNOS* gene (DBKO) exhibited a decreased (*p<*0.01) body weight accompanied by significantly (*p<*0.01) smaller epididymal, subcutaneous and total fat depots as compared to *ob/ob* mice. The weight gain of DBKO mice during the study was significantly reduced (*p*<0.0001) as compared to *ob/ob* mice (25.1±0.6 g *vs* 30.8±0.7 g). Twelve week-old DBKO mice showed a reduced (*p<*0.01) food intake (Fig 1C) exhibiting a lower food efficiency as compared to *ob/ob* mice (Fig 1D). Basal rectal temperature was analyzed showing that *iNOS* deficiency improved the reduced rectal temperature of *ob/ob* mice (*p<*0.05) (Fig 1E). In a subset of mice, a glucose tolerance test was performed. The glucose areas under the curves (AUC) were measured using the trapezoidal method. The glucose AUC in *ob/ob* mice was significantly higher (*p*<0.001) than that of wild type mice. Moreover, deletion of the *iNOS* gene significantly decreased the glucose AUC (*p*<0.05) in wild type and *ob/ob* mice (wild type 540±27, *iNOS*−*^/^*− 474±29, *ob/ob* 706±71, DBKO 618±90). As can be observed in Table 2, absence of leptin was associated with insulin resistance as evidenced by the increased concentrations of glucose, insulin, glucose AUC and HOMA index as well as by low adiponectin levels (*p<*0.01). *iNOS* deletion significantly reduced FFA and cholesterol concentrations in wild type and *ob/ob* mice (*p<*0.01). DBKO mice exhibited a tendency towards an improved insulin sensitivity as compared to *ob/ob* mice as evidenced by the reductions in insulin, HOMA index (*p* = 0.071 and *p* = 0.069, respectively) and glucose AUC.

**Table 2:** Metabolic characteristics of 12-week-old experimental animals.

	Wild type	*iNOS*−^/^−	*ob/ob*	DBKO
Glucose (mg/dl) a	75±4	79±2	105±7	99±4
Glycerol (mg/dl) a	0.022±0.002	0.023±0.003	0.034±0.001	0.032±0.002
FFA (mmol/l) a, b	0.70±0.06	0.58±0.03	1.13±0.10	0.85±0.04
TG (mg/dl) a	70±4	77±4	97±8	96±7
Cholesterol (mg/dl) a, b	83±7	71±4	177±15	158±11
Insulin (ng/ml) a, c	0.39±0.01	0.33±0.04^d^	11.20±1.54^e^	8.75±0.81e,f
HOMA a	1.78±0.22	1.55±0.18	79.45±13.93	47.27±3.14
Adiponectin (µg/ml) a	23±3	26±3	14±2	20±4

Data are means ± SEM of 8–10 animals. *P* values obtained by two-way ANOVA are shown. One way ANOVA followed by Tukey's *post hoc* tests were applied for variables with interaction between factors.

a effect of the absence of the *ob* gene (*p<*0.01);

b effect of the absence of the *iNOS* gene (*p<*0.01);

c interaction between factors (*p<*0.05);

d *p<*0.05 vs wild type;

e *p<*0.001 vs wild type;

f *p = *0.071 vs *ob/ob*. FFA: free fatty acids, HOMA: homeostasis model assessment, TG: triglycerides. DBKO: double knockout mice simultaneously lacking the *ob* and *iNOS* genes.

### Brown adipose tissue phenotype of *ob/ob* mice lacking the *iNOS* gene

**Figure 2:**
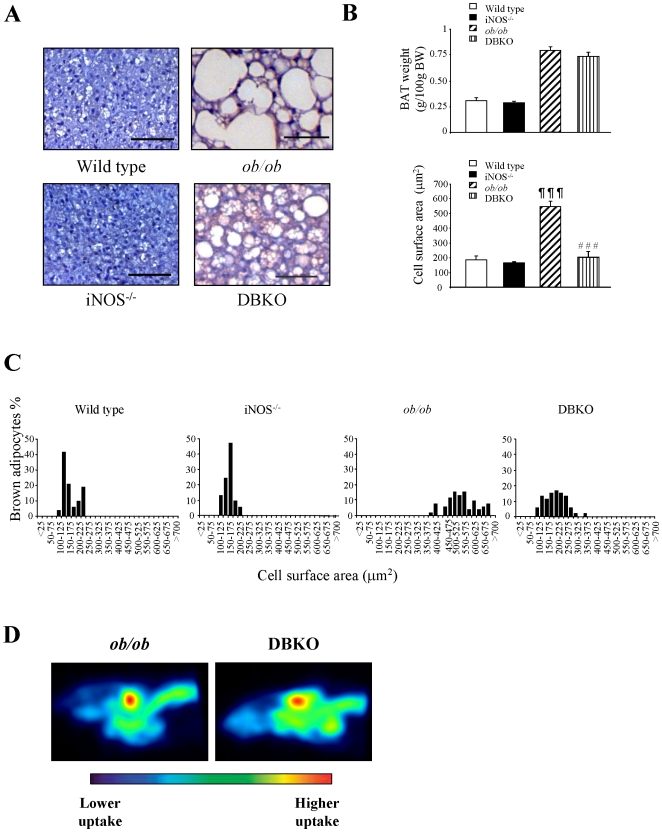
Phenotype of BAT of the experimental groups. (A) Representative histological sections of BAT stained with hematoxylin-eosin. Magnification X100 (scale bar = 50 µm). BAT weight, general cell surface area (B), and mean values (C) of the cell surface area in relation to the percentage of brown adipocytes contributing to the final cell size in each of the experimental groups. Values are the mean ± SEM (n = 6 per group). (D) MicroPET scans depicting interscapular BAT uptake of experimental animals using ^18^F-FDG as a probe; signals are shown in %ID/g at the region of interest over the background. Differences between groups were analyzed by one-way ANOVA followed by Tukey's *post hoc* test. ¶¶¶*p<*0.001 vs wild type, ###*p<*0.001 vs *ob/ob* mice.

The weight of interscapular BAT was increased (*p<*0.001) in leptin-deficient mice (wild type 0.31±0.02, *ob/ob* 0.80±0.04 g/100 g body weight). Deletion of *iNOS* slightly decreased the weight of BAT in wild type (wild type 0.31±0.02, *iNOS*−^/^− 0.29±0.01 g/100 g body weight) and *ob/ob* mice (*ob/ob* 0.80±0.04, DBKO 0.75±0.04 g/100 g body weight) although the differences did not reach statistical significance (Fig. 2B). However, the cross-sectional area of brown adipocytes of experimental animals was determined and as expected showed small and multilocular lipid droplets in control mice, whereas *ob/ob* mutants exhibited large and unilocular lipid droplets (Fig. 2A). The deletion of the *iNOS* gene in *ob/ob* mice dramatically reduced the size of brown adipocytes. In this sense, DBKO mice displayed a higher proportion of small multilocular adipocytes together with a lower proportion of larger unilocular “white-like” adipocytes as corroborated by the cell surface area (*p<*0.001) (Fig. 2C). As seen in Fig. 2D, microPET scans revealed a markedly enhanced ^18^F-FDG uptake in the interscapular BAT of DBKO mice compared to *ob/ob* animals.

### Up-regulation of brown adipocyte function markers in *ob/ob* mice lacking the *iNOS* gene

**Figure 3:**
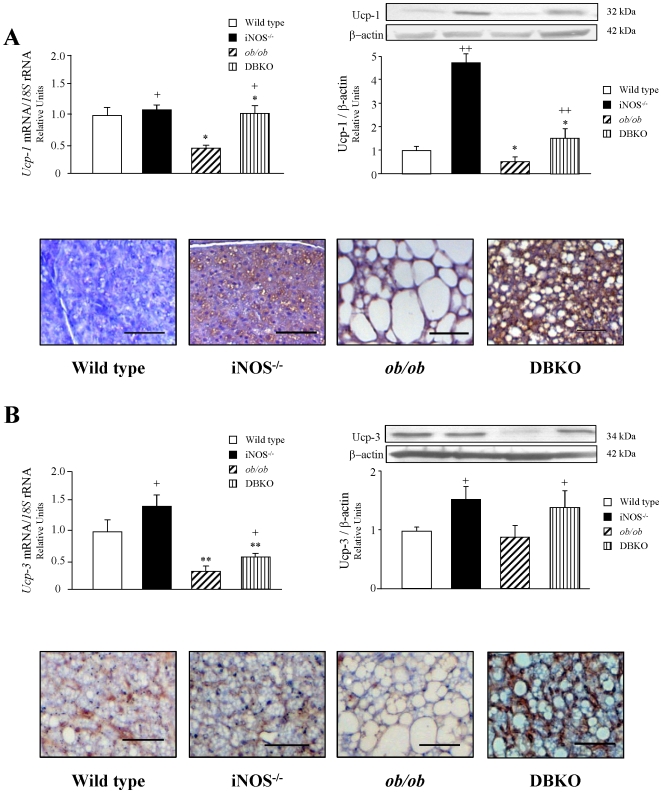
Gene and protein expression levels of genes involved in thermogenesis. Expression levels of UCP-1 (A) and UCP-3 (B) in BAT. mRNA and protein data were normalized for the expression of *18S* rRNA and β-actin, respectively. The expression in wild type mice was assumed to be 1. Representative blots are shown on top of the histograms. Immunohistochemistry of UCP-1 and UCP-3 in BAT corresponding to each experimental groups is shown at the bottom of the histograms. Magnification X100 (scale bar = 50 µm). Values are the mean±SEM (n = 6 per group). Differences between groups were analyzed by two-way ANOVA. *p<0.05, **p<0.01, effect of the absence of the *ob* gene. +*p<*0.05, ++*p<*0.01, effect of the absence of the *iNOS* gene.

The mRNA and protein expression levels of molecules involved in the regulation of thermogenesis and mitochondrial function were analyzed in BAT of the experimental animals. As shown in Fig. 3, the gene and protein expression levels of Ucp-1 and Ucp-3 were down-regulated in *ob/ob* mice and up-regulated in *iNOS-*deficient mice as compared to those of wild type mice. Noteworthy, DBKO mice simultaneously lacking the *ob* and *iNOS* genes showed a statistically significant increase in Ucp-1 (*p<*0.05) and Ucp-3 (*p<*0.05) transcripts and proteins compared to *ob/ob* mice. Immunohistological analyses showed a high expression of Ucp-1 and Ucp-3 in BAT in all experimental groups. Nonetheless, the immunostaining of both proteins was markedly increased in *iNOS* knockout and DBKO mice, and decreased in *ob/ob* animals as compared to wild types.

### *ob/ob* mice lacking *iNOS* display changes in gene expression levels of molecules involved in brown fat cell differentiation

**Figure 4:**
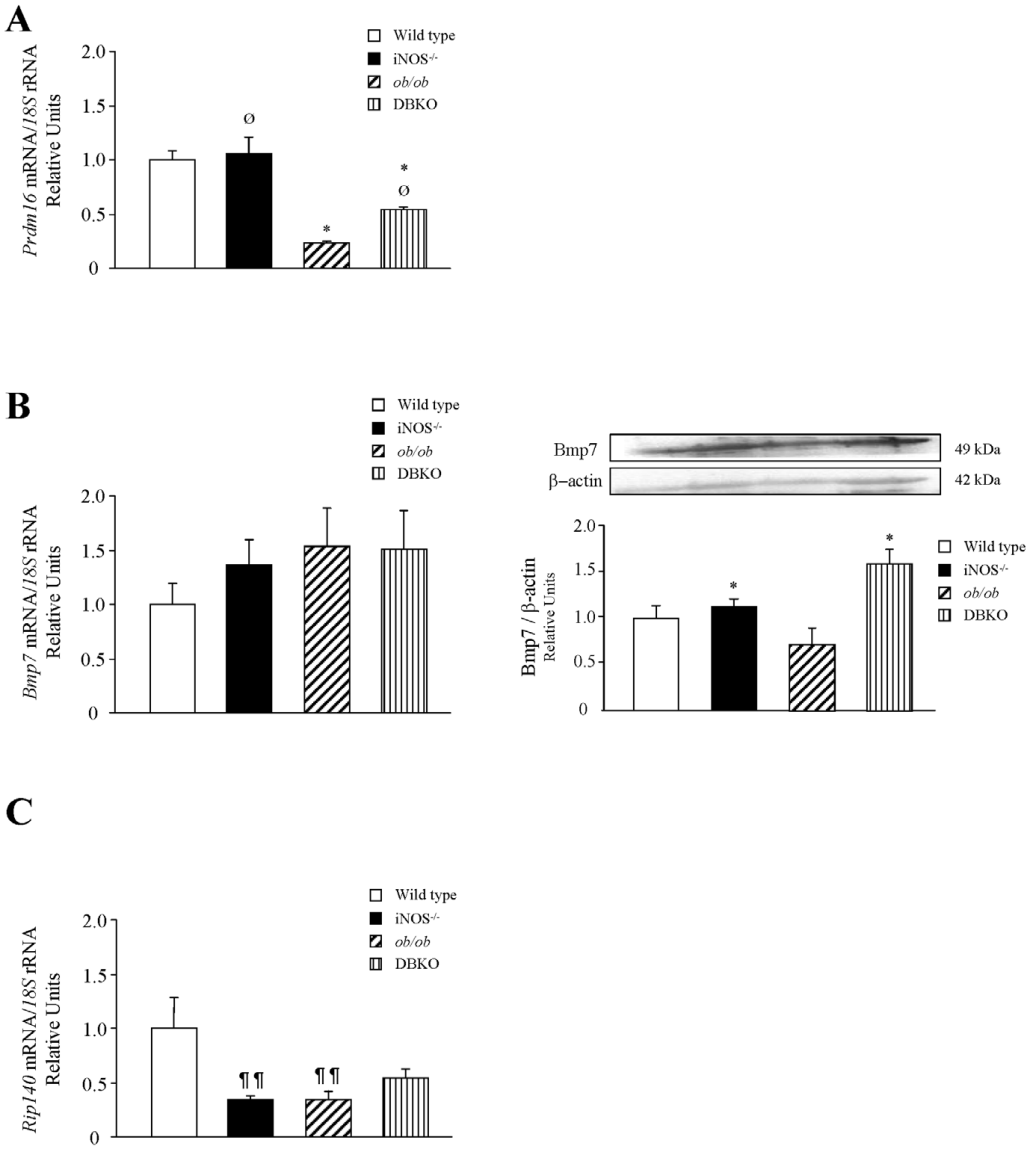
Expression of genes involved in brown fat differentiation. Gene expression levels of *Prdm16* (A), *Bmp7* (B) and *Rip140* (C). Data were normalized for the expression of *18S* rRNA and gene expression levels in wild type mice were assumed to be 1. Values are the mean ± SEM (n = 6 per group). Protein levels of Bmp7 are also shown (B). Protein data were normalized for the expression of β-actin. Differences between groups were analyzed by two-way ANOVA. *p<0.05, effect of the absence of the *ob* gene. Ø *p = *0.056, effect of the absence of the *iNOS* gene. One way ANOVA followed by Tukey's *post hoc* test was applied for variables with interaction between factors. ¶¶*p<*0.01 vs wild type.

To gain further insight into the mechanisms underlying the improved energy expenditure of DBKO mice, the gene expression levels of key molecules involved in brown fat cell differentiation were examined (Fig. 4). The gene expression levels of *Prdm16*, a zinc-finger protein that stimulates brown fat-selective gene expression, was significantly down-regulated (*p<*0.001) in *ob/ob* mice, while the deletion of *iNOS* increased mRNA expression levels although only a marginal statistical significance was found (*p = *0.056). No changes in the mRNA expression levels of *Bmp7*, a protein involved in the activation of the program of brown adipogenesis, was observed. Nevertheless, protein expression levels of Bmp7 were increased in mice lacking the *iNOS* gene. Moreover, gene expression levels of *Rip140*, a nuclear receptor involved in the differentiation of white adipocytes, was significantly decreased (*p<*0.01) in *iNOS*-deficient mice.

**Figure 5:**
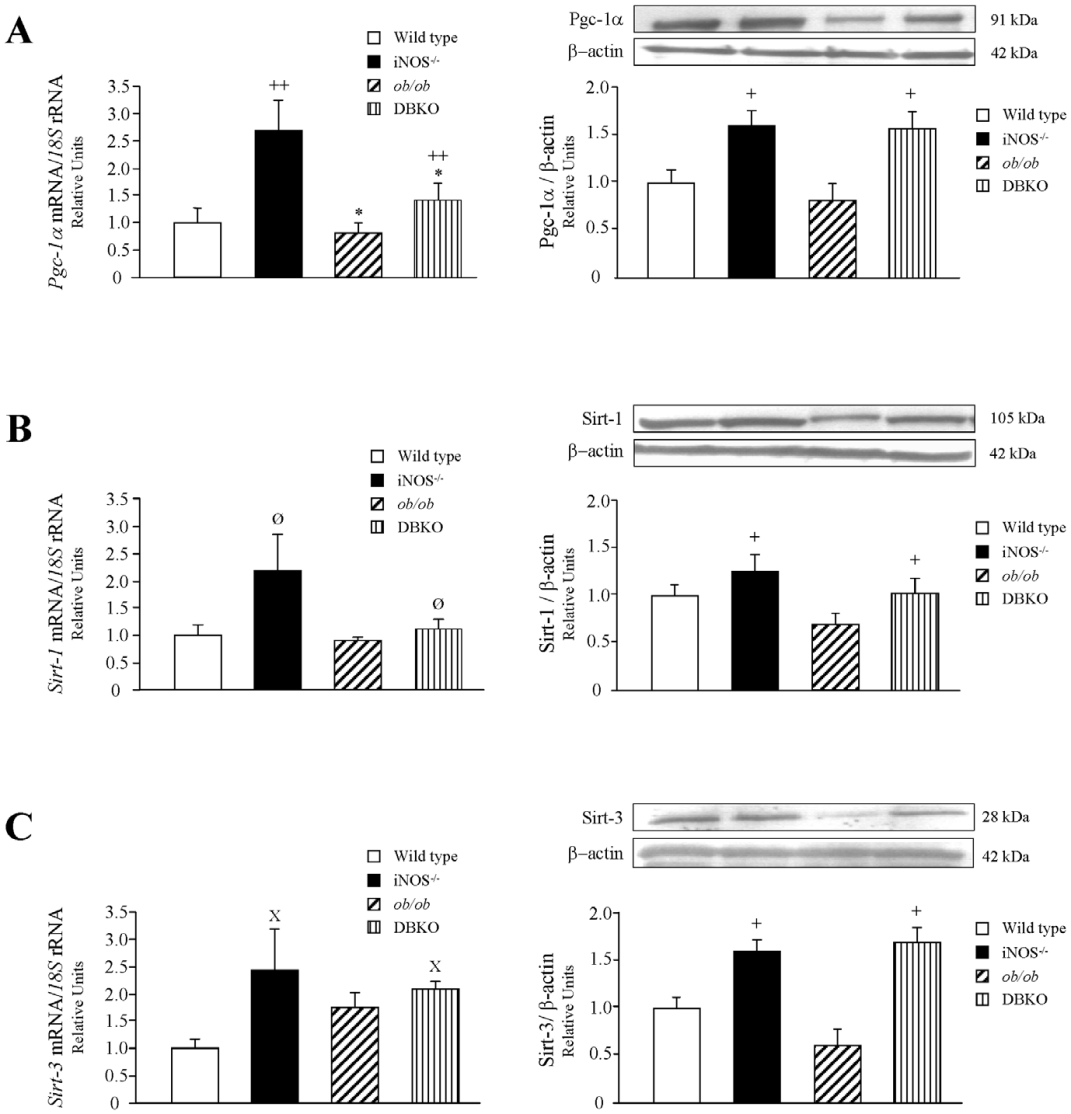
Effect of the lack of both genes on molecules involved in the regulation of thermogenesis. Bar graphs show the transcript and protein levels of peroxisome proliferator-activated γ coactivator-1 α (PGC-1α) (A), sirtuin-1 (SIRT1) (B), and sirtuin-3 (SIRT3) (C) in BAT of experimental animals. mRNA and protein data were normalized for the expression of *18S* rRNA and β-actin, respectively. The expression in wild type mice was assumed to be 1. Representative blots are shown on the top of the histograms. Values are the mean±SEM (n = 6 per group). Differences between groups were analyzed by two-way ANOVA. *p<0.05, effect of the absence of the *ob* gene. +*p<*0.05, ++*p<*0.01, effect of the absence of the *iNOS* gene. Ø *p = *0.089, effect of the absence of the *iNOS* gene; X *p = *0.084, effect of the absence of the *iNOS* gene.

The mRNA and protein expression levels of molecules involved in the regulation of mitochondrial function and thermogenesis were also analyzed. Leptin deficiency was associated with a reduction of *Pgc-1α* transcript levels, together with a tendency towards a decrease in *Sirt-1* transcript levels, without changes in the gene expression levels of *Sirt-3* (Fig. 5). On the contrary, *iNOS* knockout mice showed an increase in mRNA levels of *Pgc-1 α* at the same time as an increase in *Sirt-1* and *Sirt-3* transcript levels, although only a marginal statistical significance was found. The DBKO mice showed an up-regulation of *Pgc-1 α* (*p<*0.01) and a marginal increase in *Sirt-1* and *Sirt-3* as compared to the *ob/ob* group, although in the case of the sirtuins differences were not statistically significant. The protein expression of Pgc-1α, Sirt-1 and Sirt-3 in BAT exhibited a similar pattern to that observed in the gene expression analyses.

## Discussion

The involvement of leptin and *iNOS* in the control of energy balance through actions on food intake, body weight and energy expenditure has been previously reported [16], [33], [40]. Leptin-deficient mice exhibit marked obesity, hyperphagia, insulin resistance, hypothermia and increased food efficiency [40], whereas *iNOS* knockout mice are resistant to diet-induced obesity, showing reduced epididymal fat pads and increased body temperature [26], [33]. Although the *iNOS* deficiency did not completely restore the phenotype of the absence of leptin, our results show that deletion of the *iNOS* gene exerts a significant impact on energy homeostasis via increasing energy expenditure and decreasing food intake. We observed that the absence of leptin leads to obesity even in the context of *iNOS* deficiency. The DBKO mice showed a modest, but consistent lower body weight than that of *ob/ob* mice at the end of the study. The data shows that from the eleventh week of the study onwards DBKO mice weigh significantly less than *ob/ob* mutants. Moreover, these differences were maintained and increased from this time point throughout longer experimental periods (32 weeks, data not shown). The possibility that DBKO mice weigh less at the initial weeks of the study and therefore, the increase in body weight may be similar to that of *ob/ob* mice at the end of the experimental period is ruled out since from the beginning the DBKO exhibited a slightly higher body weight than *ob/ob* animals. Thereafter, the growth curve of *ob/ob* mice continues to increase steadily while that of DBKO slows down. Body weights of both experimental groups are superimposed from 8–10 weeks. During that period the growth curves of both groups intercross with *ob/ob* mice becoming heavier than DBKO mutants. This leads towards the end of the study to a more evident reduction in body weight meaning that the lack of *iNOS* is exerting an impact on body weight in *ob/ob* mice. The differences in body weight were attributable in part to a reduction in food intake. We describe, for the first time, that the disruption of the *iNOS* gene reduces the elevated food intake and food efficiency, partially ameliorating the obesity of *ob/ob* mice. It has been proposed that leptin-deficient mice show higher food efficiency rates at least in part due to an impaired capacity of BAT to produce heat [2], [41]. In this sense, our data confirm this observation and further show that *iNOS*-deficient and DBKO mice exhibit an increase in rectal temperature. Taken together, ablation of *iNOS* improves the energy balance of *ob/ob* mice by decreasing energy intake (reduced food intake), and by increasing energy expenditure (increased rectal temperature). It is well known that *ob/ob* mice have an increased respiratory quotient (RQ) due to a reduced fat oxidation with leptin replacement normalizing the metabolic rate in these animals. *iNOS* knockout mice probably exhibit a reduced RQ due to their increased β-oxidation in brown adipose tissue, as reflected by their reduction in circulating free fatty acids. It seems reasonable that the absence of *iNOS* may be associated to an increase in energy expenditure given that *iNOS* and DBKO mice exhibit a reduced body weight and fat mass as compared to their respective controls. This is supported by published studies [42], [43] evidencing that nitric oxide inhibits cytochrome c oxidase, thus inhibiting the mitochondrial respiratory chain. The participation of NO in thermoregulation is based on its vasodilator properties and its regulatory role in non-shivering thermogenesis. Previous studies were focused on the existence of two isoforms of NOS in BAT: eNOS and iNOS. A role for iNOS in the regulation of sympathetically-mediated blood flow in brown adipose tissue has been described, arguing against an increased energy expenditure in this tissue in *iNOS* knockout mice [23]. Furthermore, *eNOS*-deficient mice show a reduced energy expenditure and an increased body weight [44]. On the other hand, it has been reported that NO downregulates the expression of UCPs in adipocytes [45] with UCPs being heavily involved in energy expenditure regulation. The lack of iNOS may decrease NO production in adipose tissue in an autocrine/paracrine way, thereby increasing the expression of UCPs and hence energy expenditure. The observed increase in the expression of Ucp-1 and Ucp-3 in BAT of *iNOS*-deficient mice in the present study supports this mechanism. However, NO involvement in the regulation of energy expenditure is complex and may exhibit NOS-specific differences. On the other hand, *ob/ob* mice exhibit a reduced locomotor activity, which is normalized after leptin administration. The fact that the proposed rise in energy expenditure in *iNOS*-deficient mice is due to an increased locomotor activity may be excluded given the fact that NO has been reported to induce locomotor activity in mice [46] while *eNOS*-deficient mice show normal locomotor activity [44]. Regular observation of the DBKO and iNOS−^/^− mice during the whole experimental period of our study did not identify qualitive or semiquantitative changes in locomotor activity and behaviour between the different rodents. In this sense, the preponderance of an effect on body temperature in the absence of changes in locomotor activity may be put forward. Undoubtedly, the detailed analysis of the potential impact on both energy expenditure and locomotor activity with sophisticated equipment to pick up slight differences would merit a study on its own to clarify the exact contribution of each component.

The activation of BAT has been evidenced to play an important role in energy expenditure [47]. Previous studies have reported a “white-like” appearance of BAT in *ob/ob* mice, suggesting a crucial role of leptin in the development of brown adipocytes [22]. In the histological analyses, large unilocular lipid droplets were observed in BAT of leptin-deficient mice. We also showed that in the DBKO mice the characteristic features of BAT tissue (both macro and microscopically as well as molecularly) are partially restored. To gain further insight into the mechanisms underlying the change in the phenotype of BAT of the experimental models, we focused on the transcriptional control of the metabolic pathways, achieved by the coordinated actions of numerous transcription factors and associated coregulators or corepressors. In this sense, an increase in the gene expression levels of the recently identified transcription factor PRDM16, a positive transcriptional regulator of the brown fat cell gene program [10] was observed in *iNOS*-deficient mice. We also studied the expression of a member of the family of the bone morphogenetic proteins (BMPs), Bmp7, that reportedly regulates energy homeostasis by activating a full program of brown adipogenesis [48]. *iNOS* deficiency enhanced Bmp7 protein expression. Moreover, the corepressor Rip140, that plays a key role in energy homeostasis by repressing metabolic gene networks [49], was dramatically reduced in *iNOS* knockout and *ob/ob* mice. These data are in line with previous studies in humans [50], supporting the notion that downregulation of *Rip140* may be a compensatory mechanism in order to favour energy expenditure and fat accumulation reduction in already established obese states.

The expression of key markers of brown fat cell function was also investigated. Pgc-1α is an important factor in mitochondrial function and energy homeostasis, and controls several aspects of mitochondrial biogenesis. It plays an essential role in brown fat thermogenesis, trough activation of UCP-1 [51]–[53]. Sirt-1 positively acts on the activation of metabolic genes through a direct deacetylation of the transcriptional coactivator Pgc-1α. Moreover, growing evidence supports a novel role of Sirt-3 in enhancing the expression levels of mitochondria-related genes, participating in adaptive thermogenesis [54]. An upregulation of these factors that control brown fat cell function in BAT of DBKO as compared to *ob/ob* mice was detected. These results were concomitant with a significant increase in the expression of the brown adipocyte-specific gene Ucp-1 in *iNOS*-deficient mice. It is well known that Pgc-1α is intimately involved in adaptive thermogenesis via the induction of the mitochondrial inner membrane uncoupling protein Ucp-1 [55]. Ucp-3 is another member of the uncoupling proteins family located in BAT [56]. It has been recently shown that, in addition to participating in lipid metabolism and defense against reactive oxygen species, Ucp-3 is also implicated in thermogenesis, and its absence is associated with impaired cold tolerance and decreases expression of metabolic genes [57]. In this sense, levels of UCP-3 were reduced in the absence of leptin and were significantly upregulated by the deficiency of the *iNOS* gene. Further *in vitro* studies would help to confirm the effect of iNOS on brown adipocyte differentiation.

Our data reveals that *iNOS* ablation improves the brown-like phenotype and the molecular function of brown fat in *ob/ob* mice, thus improving the energy balance and increasing the thermogenic activity of these animals. Interestingly, mitochondrial NOS (mtNOS) has been shown to modulate bioenergetics regulating oxygen uptake by reversible inhibition of cytochrome oxidase [58]. Thus, NO produced by mtNOS is involved in setting the oxygen uptake level in the cell as a metabolic adaptation. In the setting of *iNOS* deficiency a potential compensatory upregulation of the mtNOS isoform may take place. From a teleological point of view the coupling of thermogenesis with the metabolic response during infection via the different NOS isoforms is justified to warrant an adequate immunologic response at the same time as avoiding an exaggerated thermogenic effect in a catabolic setting. An iNOS-induced NO production after an infection has been shown to be an important mediator of the febrile response. Previous studies [59] have reported that *iNOS* knockout mice respond with lower fever after LPS administration. However, the febrile response and non-shivering thermogenesis are mechanistically different. The present study does not allow to conclude whether the observed effects are due to an independent effect of leptin and NO via the sympathetic nervous system or due to an interaction between the signalling cascades of both molecules. Noteworthy, several interactions in different physiological systems have been further described. NO is involved in the effects of leptin and neuropeptide Y on food intake, as well as in other biological actions, such as glucose [26] and lipid [28] homeostasis, vascular tone regulation [29], [60], [61], reproduction [62] or immune response [33], [63]. Consistent with its pleiotropic role, leptin interacts with many signalling pathways including those involving NO [64]. A functional relation between leptin and NO in many cell types and biological processes has been established. In this context, our group was the first one to identify that NO represents the key molecule for the depressor response induced by leptin in the control of blood pressure [27] via iNOS-mediated signalling [29]. Interestingly, leptin was shown to play a dual role on blood pressure, whereby it increased arterial pressure through its sympathoexcitatory activity at the same time as exerting a depressor response attributable to NO release. Therefore, it is foreseeable that the inhibition of NOS leads to a predominant effect on the sympathetic activation.

Obesity is characterized by a low-grade chronic inflammatory state; it causes the activation of an inflammatory process in metabolically active sites such as adipose tissue, liver or immune cells and the altered production of immunomodulators and pro-inflammatory molecules that contribute to the induction of iNOS [26], [65]. This enzyme plays a crucial role against microbial pathogens and tumor cells, immunopathologies and in immune regulation, since NO contributes to local immune defence during inflammatory processes. In this sense, it has been reported that mice lacking *iNOS* are more susceptible to viral infections [66]. Moreover, the obese state is associated with alterations in immune function since obesity interferes with the ability of the immune system to appropriately respond to infections [67]. Furthermore, several studies suggest that iNOS induction is involved in cytokine-induced insulin resistance, given that increased iNOS expression is related to an impaired insulin-stimulated glucose uptake [68] revealing a profound involvement of iNOS in both immunologic and metabolic systems. In this line, the present study shows that ablation of the *iNOS* gene in *ob/ob* mice improves the impaired carbohydrate metabolism, as previously described [69], [70], decreasing glycemia and insulinemia as well as increasing adiponectinemia, at the same time as it ameliorates lipid homeostasis through a decrease in serum triglycerides and total cholesterol levels.

Taken together, deletion of the *iNOS* gene improves the brown-like phenotype and the molecular function of brown fat in *ob/ob* mice. *iNOS* deficiency results in decreased body weight and reduced white fat pads, not only in wild type but also in leptin-deficient obese mice. The anti-obesity effect of the absence of *iNOS* is probably due to changes in pathways promoting the differentiation of brown fat cells, and changes in genes involved in brown fat function, such as Sirt1, Sirt-3 and Pgc-1α. Moreover, ablation of *iNOS* increases Ucp-1 and Ucp-3 expression, which, in turn, may increase the rate of β-oxidation, leading to the increased consumption of FFA in BAT as fuel for adaptive thermogenesis. These data suggest that attenuated adiposity and improved energy expenditure in *iNOS*-deficient wild type and *ob/ob* mice are functionally related to increased thermogenesis in BAT.
